# Determination of the Infection Dynamics of *Escherichia coli* O157:H7 by Bacteriophage ΦV10

**DOI:** 10.3390/foods14040617

**Published:** 2025-02-13

**Authors:** Michael F. Oats, Claudia P. Coronel-Aguilera, Bruce M. Applegate, Laszlo N. Csonka, Arun K. Bhunia, Andrew G. Gehring, George C. Paoli

**Affiliations:** 1 Department of Biological Sciences, Purdue University, West Lafayette, IN 47907, USAcsonka@purdue.edu (L.N.C.); 2 Department of Food Science, Purdue University, West Lafayette, IN 47907, USA; 3 Purdue University Interdisciplinary Life Science Program (PULSe), Purdue University, West Lafayette, IN 47907, USA; 4 Department of Comparative Pathobiology, Purdue University, West Lafayette, IN 47907, USA; 5 Characterization and Interventions for Foodborne Pathogens Research Unit, Eastern Regional Research Center, Agricultural Research Service, U.S. Department of Agriculture, Wyndmoor, PA 19038, USA

**Keywords:** bacteriophage, *E. coli* O157:H7, ΦV10, phage binding

## Abstract

ΦV10 is an *Escherichia coli* O157:H7-specific bacteriophage that has been used to develop luminescent reporter assays for the detection of this important foodborne pathogen. Previous work demonstrated the specificity of ΦV10 for infection of *E.coli* O157:H7 through interaction with the O157 antigen. In addition, modification of the lipopolysaccharide (LPS) via O-acetylation prevents ΦV10 infection in an *E. coli* O157:H7 expressing a phage-encoded O-acetylase gene. Through assays for phage binding, plaque formation, and lysogeny using non-O157:H7 and O157: non-H7 strains, as well as complementation of an O157:H^−^ strain, it is demonstrated in this study that both the somatic O157 antigen and flagellar H7 antigen are required for productive infection of *E. coli* O157:H7 by ΦV10. Together, the results indicate that the O157 antigen is required for phage binding and that the H7 antigen is necessary to complete the infection process.

## 1. Introduction

Shiga toxin-producing *Escherichia coli* (STEC) O157:H7 is an important human pathogen, causing approximately 63,000 cases of foodborne illness in the United States and 2.8 million cases worldwide every year [1]. STEC O157:H7 pathogenesis begins with infection of the host intestines, causing attachment effacement lesions in the gut wall [2]. It can induce cramping, diarrhea (sometimes bloody), and colitis, resulting in significant fluid loss. However, Shiga toxin production can also induce a potentially fatal condition known as hemolytic uremic syndrome (HUS), in which severe and potentially life-threatening damage can occur to the kidneys. HUS occurs in approximately 10% of *E. coli* O157:H7 infections, with a mortality of approximately 5% [1]. This underscores a critical need to detect *E. coli* O157:H7 contamination in food before it reaches consumers [3].

In addition to the health consequences that *E. coli* O157:H7 can induce in individuals and populations, it can also have a widespread economic impact. For example, a 2018 outbreak in Romaine lettuce is estimated to have caused USD 69.4 million in combined damages to retailers and producers [4]. As a whole, *E. coli* O157:H7 infections result in approximately USD 255 million in losses every year in the United States [5]. This demonstrates not only a critical need to detect *E. coli* O157:H7 before it reaches consumers for healthcare reasons, but it also indicates that there can be economic benefits as well.

Foodborne pathogen detection methods can take many different forms, ranging from traditional microbiological and biochemical testing to more advanced immunological and nucleic acid-based methods [6]. Examples of a variety of detection approaches specific to *E. coli* O157:H7 can be found in Table 1.

Additional methods have also been developed that leverage the natural specificity of bacteriophages [19]. A variety of detection techniques have been developed using bacteriophages to detect foodborne pathogens. These techniques include biosensors, phage immobilization, and other phage-mediated bacterial detection techniques [19].

Phage ΦV10 is a temperate bacteriophage that belongs to the *Podoviridae* family. ΦV10 was used with several other phages in a phage typing scheme for strains of STEC serotype O157:H7 [20] and has been developed into a bioluminescent reporter for *E. coli* O157:H7 [21,22]. We previously reported the construction of a ΦV10 NanoLuc^®^ (*nluc*) luciferase-based (Promega, Madison, WI, USA) reporter phage by cloning the luciferase gene from *Oplophorus gracilirostris* into the ΦV10 genome, producing a ΦV10 variant known as ΦV10*nluc* [22]. NanoLuc^®^ luciferase is an enzyme that upon exposure to a commercial luciferin substrate (known as Nano-Glo^®^) emits a bioluminescent signal that can be detected using a luminometer. This bioluminescent signal is amplified if successful infection occurs due to the propagation of ΦV10*nluc*. One of the advantages of using ΦV10*nluc* is that it is specific for *E. coli* O157:H7, making it useful for phage-based detection of this foodborne pathogen [22]. In addition, previous research has demonstrated that the ΦV10*nluc* reporter construct forms lysogens at a high frequency, providing an additional advantage for facile pathogen detection [22]. ΦV10*nluc* has been used to detect *E. coli* O157:H7 in ground beef, demonstrating that it has applications in the food industry [22].

One key feature of ΦV10 is that infection of STEC O157:H7 seems to require the presence of both the O157 and H7 antigens. The lack of either the O157 or H7 antigen resulted in the absence of visible plaque formation when bacterial strains were exposed to ΦV10 [22]. Other research has demonstrated that not only is the O157 antigen a key part of the infection process but infection by ΦV10 results in the modification of the O157 antigen [23]. The ΦV10 genome contains an O-acetyltransferase gene whose product modifies the O157 antigen. The introduction of the O-acetyltransferase gene into a wild-type *E. coli* O157:H7 strain resulted in the modification of the O157 antigen that confers immunity to infection by ΦV10 [23]. Acetylation of the O antigen resulted in no plaque formation, providing evidence that not only was the O157 antigen important for infection, but O157 also appears to be a binding site for ΦV10 [23]. This indicates that the presence of a ΦV10 prophage prevents the binding of ΦV10 to the O157 antigen and superinfection of *E. coli* O157:H7 by ΦV1 phages. This exclusion mechanism could prevent the detection of *E. coli* O157:H7 containing a ΦV10 prophage.

This study focuses on the requirement of temperate bacteriophage ΦV10 for the H7 flagellar antigen as well as the somatic O157 antigen for the infection of STEC O157:H7. The purpose of this study was to determine the binding sites of ΦV10 in regard to infecting *E. coli* O157:H7, with the intent of obtaining a greater understanding of the infection dynamics of ΦV10 and *E. coli* O157:H7. Understanding these specific interactions for successful ΦV10 infection of *E. coli* O157:H7 could potentially allow a synthetic biology approach to alter the host range of ΦV10*nluc*. This could lead to a suite of biosensors for the detection of STEC serovars and allow multiplexing for simultaneous detection.

## 2. Materials and Methods

### 2.1. Strains and Growth Conditions

The *E. coli* strains, bacteriophage, and plasmids used in this study are listed and described in Table 2. Unless noted otherwise, bacterial strains were grown in lysogeny broth (LB) (10 g/L of tryptone, 5 g/L of yeast extract, and 10 g/L of sodium chloride) at 37 °C.

### 2.2. Bacteriophage Plaque Assays

Standard soft agar overlay plaque assays were conducted to enumerate plaque formation when ΦV10 was exposed to different bacterial strains [24]. In brief, the first step was to inoculate LB with a strain of interest and to grow overnight at 37 °C. The following day, 4 mL aliquots of a 0.6% LB top agar solution were placed in a steamer to melt the agar for approximately 30 min before being placed in a 40–42 °C water bath. Next, 200 µL of a bacteria strain suspension and 100 µL of a phage dilution were combined, mixed in a tube, and poured onto an LB plate, containing an antibiotic if necessary. The plates were then incubated at 37 °C overnight (~18 h). The following day, the plates were analyzed to count plaques and determine the phage titer. *E. coli* O157:H7 strain C7927 was used for the propagation of ΦV10 and generally used for phage titering.

**Table 2:** List of *E. coli* strains, bacteriophage, and plasmids used in this study.

*E. coli* Strain, Phage, or Plasmid	Description	Source
*E. coli* Strain Name		
*E. coli* C7927	Serotype O157:H7 human isolate, host cell for the propagation of ΦV10 and ΦV10*nluc*	[25]
*E. coli* 493-89	Serotype O157:H^−^ strain with a 12 bp deletion in the *flhC* flagellar regulatory gene and an intact *fliC*_H7_ gene	[26]
*E. coli* O157:H7 OAC	Strain C7927 containing pFSP177	This study
*E. coli* K12	Non-pathogenic *E. coli*	ATCC
*E. coli* EDL933	Serotype O157:H7; source of *fliC* gene	[27]
*E. coli* TOP10	General *E. coli* cloning host	ThermoFisher
*E. coli* P11	O157:H16 (O157:H7)	G.C. Paoli
*E. coli* 1880	O55:H7 (non-O157:H7)	G.C. Paoli
Phage Name		
ΦV10	Wild-type *E. coli* O157:H7-specific phage	Lab collection
ΦV10nluc	ΦV10 containing NanoLuc luciferase gene; kanamycin resistance	[22]
Plasmid Name		
pGEM^®^-T Easy	General cloning vector; ampicillin resistance	Promega
pCR2.1-TOPO	General cloning vector; ampicillin and kanamycin resistance	ThermoFisher
pGEM^®^-T Easy *flhDC*	pGEM^®^-T Easy vector containing a wild-type *flhDC* operon from *E. coli* O157:H7 C7927	This study
pFSP177	pBAD TOPO TA plasmid containing the ΦV10 O-acetyltransferase gene	[23]
pCR2.1-FliC	pCR2.1-TOPO with the cloned *fliC*_H7_	This study
pFSP182	pCR2.1 with wild-type *flhDC* and *fliC*; kanamycin and ampicillin resistance	This study

### 2.3. Generation of E. coli O157:H^−^ 493-89 Containing pFSP177 (pBAD Plasmid Expressing O-Acetyltransferase)

*E. coli* O157:H7 OAC (*E. coli* C7927 containing pFSP177 was grown overnight at 37 °C in LB with antibiotic selection (ampicillin 100 μg/mL). The pFSP177 plasmid was then isolated from *E. coli* O157:H7 OAC via the use of the Wizard^®^ Plus SV Minipreps DNA Purification System from Promega according to the manufacturer’s protocol (Promega, Madison WI, USA). The plasmid was then electroporated into previously prepared electrocompetent *E. coli* O157:H^−^ 493-89 using a BTX ECM 399 electroporator (Genetronics Inc., San Diego, CA, USA) using a 5 ms pulse of 1250 V with 1 mm gapped cuvettes. The cells were then allowed to recover by incubation with 900 μL of super optimal broth with catabolite repression media (SOC; 2 g/L tryptone, 0.5 g yeast extract, 1 mL of 1 M NaCl, 0.25 mL of 1 M KCl) for an hour and a half at 37 °C. The cells were then plated onto LB-ampicillin plates (100 μg/mL) and incubated overnight at 37 °C. A positive transformant was isolated for further study.

### 2.4. Bacteriophage Binding Assays

Bacteriophage binding assays were used to determine if ΦV10 could bind to different strains of interest. In brief, the first step was to inoculate separate LB broths with different *E. coli* strains to be tested. *E. coli* K12 was used as a negative control strain and *E. coli* O157:H7 C7927 as a positive control. The strains were grown at 37 °C for ~18 h. The optical density (OD) of the culture was measured at 600 nm. The OD600 of the cultures was adjusted to between 0.4 and 0.5 for use in the binding assays. Nine hundred microliters of the OD-adjusted culture of each strain (~10^9^ CFU/mL) were tested using 10^4^ PFU of phage (100 μL of 10^5^ PFU/mL). After mixing, the bacteria–phage solutions were left at room temperature for 10 min. The cell-bound phages were separated from unbound phages by filtration through a 0.22-micron filter. The unbound phage in the filtrate was subsequently diluted 10- and 100-fold. The titer of the diluted phage solutions was determined using *E. coli* O157:H7 C7927 as described above. Decreases in phage titers indicated phage binding.

### 2.5. Bacteriophage Lysogen Assays

Bacterial strains were grown overnight at 37 °C in LB, and the OD600 nm was adjusted to approximately 2.5. Subsequently, 900 μL of each strain were incubated with 100 μL of ΦV10*nluc* (10^6^ PFU), a modified ΦV10 bacteriophage containing a kanamycin resistance gene and the gene encoding the NanoLuc^®^ luciferase [10]. After incubation for 20 min at room temperature (20–21 °C), the mixture was serially diluted (10^−2^ to 10^−5^) and plated onto LB kanamycin (50 μg/mL) plates and grown overnight (~18 h) at 37 °C. The number of CFUs of each strain was then counted for subsequent calculation of the number of lysogens.

### 2.6. Bacterial Motility Assays

Strain motility was determined using a protocol modified from Morales-Soto (1983) [28]. Briefly, LB was inoculated with the respective strains and grown at room temperature (20–21 °C) for 48 h without shaking. Soft agar plates (1% tryptone, 0.5% sodium chloride, and 0.3% agar) were prepared and used the same day. Plates were placed in a laminar flow hood to solidify prior to inoculation. Plates were then inoculated in the center with the bacteria cultures using a toothpick and were allowed to incubate at room (20–21 °C) temperature for 24 h, examined for motility, and photographed.

### 2.7. Testing Restriction Enzyme Systems of E. coli O157:H7 C7927 and E. coli O157:H^−^ 493-89

An *E. coli* O157:H^−^ strain 493-89 ΦV10*nluc* lysogen was grown in 100 mL of LB broth containing kanamycin (50 μg/mL) at 37 °C overnight (~18 h). The entire culture was then spun down at 10,000× *g* for 10 min to separate the bacteria from the bacteriophages. The supernatant was then passed through a 0.2-micron filter (VWR International, Radnor, PA, USA). The isolated phages were then serially diluted, and plaque assays were performed with *E. coli* O157:H7 C7927 and *E. coli* O157:H^−^ 493-89.

### 2.8. Agglutination Assays

Agglutination assays were performed to verify the serotypes of the strains used in this study. The O157 and H7 antigens were assayed using the Remel^TM^ RIM *E. coli* O157:H7 Latex Test from Thermo Fisher Scientific (Thermo Fisher, Waltham, MA, USA) following manufacturer’s protocol.

### 2.9. Complementation of the flhDC Mutation in E. coli O157:H^−^ 493-89

*E. coli* O157:H^−^ strain 493-89 contains a 12-pair deletion in the *flhC* motility master regulatory gene (*flhDC*). Though the strain is considered H^−^, it contains an intact gene (*fliC*_H7_) for the H7 flagellar antigen. To complement this defect in strain 493-89, the *flhDC* genes were amplified from *E. coli* O157:H7 C7927 and cloned into pGEM^®^-T Easy. The first step in the construction of the complementary plasmid was to use polymerase chain reaction (PCR) to amplify the intact *flhDC* operon from *E. coli* O157:H7 C7927 using primers similar to those used by Monday, et al. [14] but modified to contain *Eco*RI sites. The primers used were flhDCModF, 5′-ACCTAGGAATTCGATCTGCATCACGCATTATTG-3′, and flhDCModR, 5′-ACCTAGGAATTCACTGTACCGAGAACAACCAGG-3′. The thermocycling protocol for the PCR was an initial denaturation (95 °C for 5 min) followed by 35 cycles comprising a denaturing step (95 °C for 30 s), an annealing step (60 °C for 30 s), and an elongation step (72 °C for 90 s). This was followed by a final elongation period (72 °C for 10 min) and an indefinite cooldown step to 4 °C. The pGEM^®^-T Easy Vector System from Promega (Promega, Madison, WI, USA) was used to clone the amplified fragment containing the *flhDC* operon using the manufacturer’s protocol. Plasmids containing inserts were then isolated via the Wizard^®^ Plus SV Minipreps DNA Purification System from Promega (Promega, Madison, WI, USA). Purified plasmids were then cut with *Eco*RI to check for the insert. The plasmids were then sent to the Low Throughput DNA Sequencing Laboratory at Purdue University for further confirmation of the *flhDC* operon and orientation. Plasmids that were confirmed to have the *flhDC* operon in the proper orientation were then transformed into previously prepared electrocompetent *E. coli* O157:H^−^ 493-89 via electroporation as described above. The cells were then incubated statically for 90 min at 37 °C after the addition of 900 μL of SOC medium. The cells were then plated onto LB ampicillin plates (100 ug/mL) and were grown overnight at 37 °C.

### 2.10. Construction of pCR2.1-FliC

To construct pCR2.1-FliC, the *fliC* gene was first amplified from *E. coli* O157:H7 EDL933 using primers EDL933 FliC1 (5′-GTTACAACGATTAACCCTGCAGC-3′) and EDL933 FliC2 (5′-CCCAATACGTAATCAACGACTTGC-3′). The thermocycling protocol for the PCR was an initial denaturation (95 °C for 5 min) followed by 35 cycles comprising a denaturing step (95 °C for 30 s), an annealing step (61 °C for 30 s), and an elongation step (72 °C for 90 s), subsequently followed by a final elongation period (72 °C for 1 min) and an indefinite cooldown step to 4 °C. The PCR product was then ligated to pCR2.1-TOPO and electroporated into *E. coli* TOP10.

### 2.11. Construction of pFSP182

**Figure 1:**
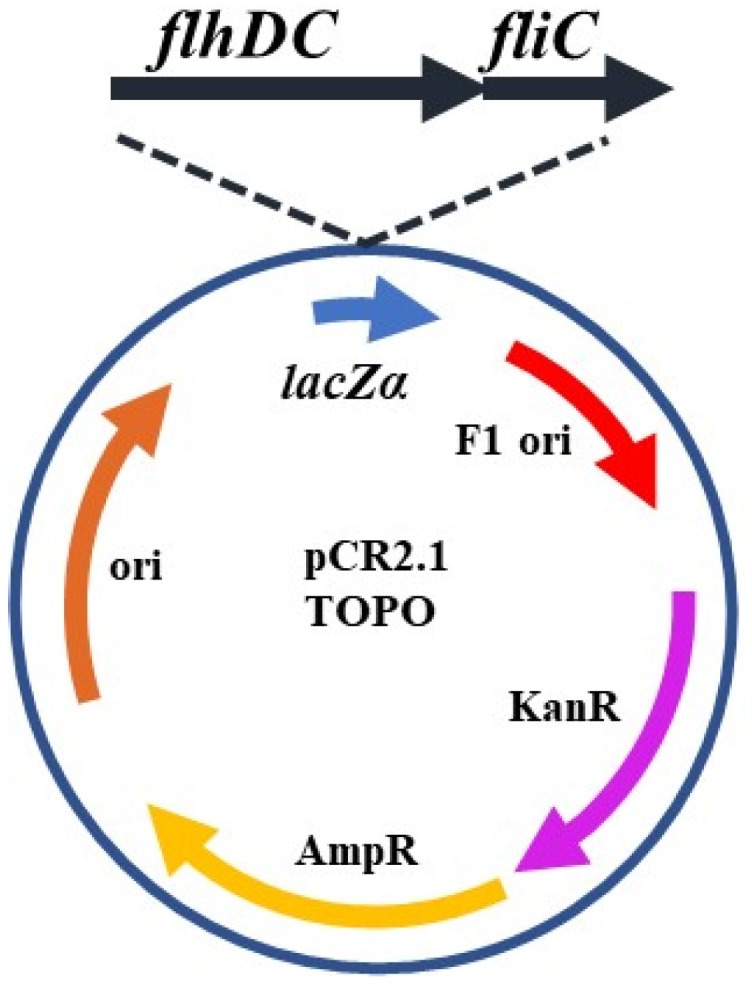
Schematic representation of pFSP182. Cloning vector pCR2.1 TOPO containing an *flhDC* gene cassette from *E. coli* O157:H7 C7927 expressed from the *lac* promoter with a downstream *fliC* gene cloned from *E. coli* O157:H7 EDL933.

The *flhDC* operon was cloned upstream of *fliC* into pCR2.1 to generate pFSP182 (Figure 1). Since the *flhDC* operon is the flagellar master regulator whose activation is required for flagellar synthesis to occur, placing it in front of the *fliC* gene should ensure proper transcription of the *fliC* gene. The pGEM-T Easy plasmid was digested with *Eco*RI and *Spe*I and plasmid pCR2.1-FliC was digested with *Xba*I and *Eco*RI. Digestion with *Spe*I and *Xba*I leaves identical single-stranded overhangs. This was useful since it would ensure that the *flhDC* operon would be inserted into the plasmid in the proper orientation for expression from the *lac* promoter. The *flhDC* operon was then ligated to the pCR2.1-FliC plasmid with T4 ligase. To confirm the orientation of the *flhDC* insert, isolated plasmids were sequenced by the Purdue University Genomics Core Facility. After confirming the proper orientation of the *flhDC* operon, the plasmid was transformed into *E. coli* O157:H^−^ 493-89 by electroporation as described above.

## 3. Results and Discussion

In previous studies, screening of numerous *E. coli* O157:H7 and strains of other *E. coli* serotypes confirmed the specificity of ΦV10 for *E. coli* O157:H7 [22,23]. Additional studies with *E. coli* O157: non-H7 and *E. coli* non-O157:H7 strains demonstrated that the lack of either the O157 or the H7 antigen resulted in no plaque formation [22]. These results indicated that ΦV10 required the presence of both the O157 and the H7 antigen to successfully infect the host cell.

While previous experiments clearly demonstrated that both the O157 and H7 antigens were required for plaque formation by ΦV10, additional experiments were conducted to more clearly determine if both antigens are required for ΦV10 binding to *E. coli* O157:H7. Previous research has indicated that the O157 antigen was critical for ΦV10 binding to occur [23]. To further define the need and role for both the O157 somatic antigen and the H7 flagellar antigen for ΦV10 infection of *E. coli* O157:H7, a series of experiments were conducted with an *E. coli* O157:H7 strain (C7927) and an *E. coli* O157:H^−^ strain (493-89).

**Figure 2:**
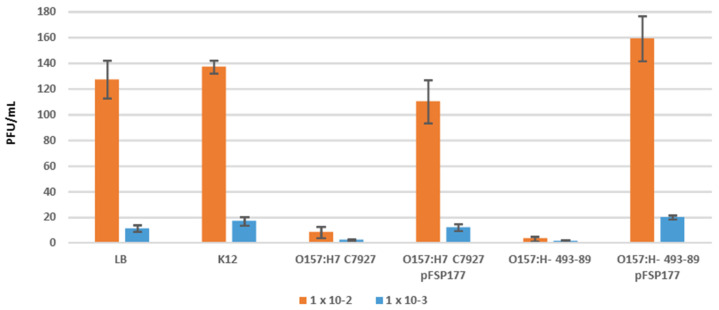
Binding assay results of *E. coli* K12, *E. coli* O157:H7 C7927, *E. coli* O157:H7 C7927 pFSP177 (pBAD OAC), *E. coli* O157:H^−^ 493-89, and *E. coli* O157:H^−^ 493-89 pFSP177 (pBAD OAC). Assays were performed in triplicate. Orange and blue bars indicate 100-fold or 1000-fold dilutions of the unbound PFU in the filtrate (unbound phage), respectively.

Plaque assays clearly indicate that ΦV10 can form plaques with *E. coli* O157:H7 strain C7927 (Table 3), but no plaques were observed when *E. coli* O157:H^−^ strain 493-89 was used in the same titer assay, even at high phage concentrations (i.e., lower dilutions; Table 3). The results reported in Table 3, the absence of plaques with *E. coli* O157:H^−^ strain 493-89, were consistently reproduced in numerous additional titer assays using several different preparations of ΦV10 that yielded high titers with strain C7927. Even though the O157:H^−^ strain 493-89 did not form plaques, phage binding assays revealed that ΦV10 is able to bind to the strain (Figure 2). In this experiment, the negative control strain *E. coli* K12 did not bind ΦV10 while, as expected, C7927 did bind ΦV10. When both C7927 and 493-89 were transformed with a plasmid pFSP177 (pBAD OAC) containing the ΦV10 *oac* gene whose product acetylates the O antigen and prevents plaque formation by ΦV10 [23], binding of ΦV10 was significantly reduced relative to the untransformed strains (Figure 2). These combined results of the assays for plaque formation and phage binding demonstrate that ΦV10 requires the O157 antigen to bind to the host cell and suggest that the H antigen is required for infection and subsequent plaque formation.

Lysogen assays were also conducted to determine if ΦV10*nluc* was able to infect *E. coli* O157:H^−^ 493-89. Lysogen assays are more sensitive than plaque assays because plaque formation requires multiple infective events to produce a visible plaque, while lysogen formation requires only one infective event to produce a positive result (a luminescent colony selected for kanamycin resistance). Four *E. coli* strains were tested for lysogen formation, O157:H7 strain C7927, O157:H strain 493-89, O157:H16 strain P11 (an O157:non-H7 strain), and O55:H7 strain 1880 (a non-O157:H7 strain). Strains *E. coli* O157:H7 C7927 and *E. coli* O157:H^−^ 493-89 formed lysogens, while *E. coli* O157:H16 P11 and *E. coli* O55:H7 1880 did not (Table 4). The lack of lysogen formation in *E. coli* O157:H16 P11 and *E. coli* O55:H7 1880 further demonstrated that both O157 and H7 are required for successful ΦV10 infection to occur. A comparison of lysogen formation by *E. coli* O157:H7 C7927 and *E. coli* O157:H^−^ 493-89 indicated that even though ΦV10*nluc* was able to infect *E. coli* O157:H^−^ 493-89, it did so at approximately 100-fold less efficiency (Table 4). Though the mechanism by which the H7 functions during ΦV10 infection and the reason ΦV10 is able to lysogenize H^−^ strain 493-89 were not specifically explored in this study, the latter may be due to the expression of the H7 flagella in a small sub-population of cells in cultures of 493-89. Briefly, the mutation resulting in the H^−^ phenotype in strain 493-89 is within the *flhC* gene, a transcriptional activator of genes required for the biosynthesis of flagella. Furthermore, the strain contains an intact *fliC*_H7_ gene. As mentioned above, lysogen formation can occur with a single phage infection, while plaque formation requires lytic infection of numerous cells. Infection by ΦV10*nluc* could result in either lysogenic or lytic infection in a small sub-population of cells in a culture of strain 493-89 that has regained the expression of H7 flagella. In our experiments, lysogens were selected using the kanamycin resistance encoded in the ΦV10*nluc*, but plaque formation would require lytic infections of numerous adjacent flagella-expressing cells on the plate, sufficient to result in a zone of clearing.

One reason for the reduced lysogen formation in strain 493-89 could be due to the presence of a restriction system in this strain. Restriction systems found in bacteria act as defenses against bacteriophages. Restriction systems work by chopping up foreign DNA. The bacteria protect their own DNA by methylating. Infection by non-methylated bacteriophage DNA could result in reduced lysogen formation. The differences in lysogen formation could be explained if strain 493-89 has a restriction system that is absent in C7927. However, bacteriophages can protect their own DNA from restriction through methylation. In this case, successful lysogeny of 493-89 by ΦV10*nluc* would result in methylation of the bacteriophage DNA. Nevertheless, when ΦV10*nluc* was isolated from a 493-89 lysogen, the resulting phage did not form plaques on a naïve strain of 493-89. These results indicate that restrictions do not play a role in ΦV10’s inability to plaque 493-89.

The results of phage binding, plaque, and lysogen assays strongly suggest that the O157 antigen is the primary receptor for ΦV10, while the flagella expressing the H7 antigen serve a secondary function for successful infection (Table 4). To further buttress the results suggesting that the H7 antigen is required for the successful infection of *E. coli* O157:H7 strains by ΦV10, experiments were carried out to determine if complementation of the *flhC* mutation in strain 493-89 by a wild-type *flhDC* operon could restore plaque formation. Initial experiments revealed that a cloned *flhDC* restored cellular motility to strain 493-89 but not plaque formation by ΦV10 (Table 5). Complementation of *E. coli* O157:H^−^ strain 493-89 to plaque formation required both the *flhDC* and *fliC*_H7_ genes in pFSP182 and resulted in cellular motility and plaque formation at a significantly lower level when compared to *E. coli* O157:H7 C7927 (Table 5). Lack of plaque formation in strain 439-89 complemented with *flhDC* alone could be the result of minor amino acid sequence variation in the 439-89 FliC, while the reduced level of plaque formation in strain 493-89 pFSP182 is likely due to suboptimal regulation and expression of the *fliC*_H7_ gene in this construct.

## 4. Conclusions

The key finding of this study was that *E. coli* O157:H7-specific bacteriophage ΦV10 requires the presence of both the O157 and H7 antigens to infect the host cell. This is significant because it demonstrates the process by which infection of the host by ΦV10 occurs. It seems that the O157 antigen serves as the binding (docking) site, while the H7 flagellar antigen is critical for subsequent infection (i.e., phage DNA entry into the host cell). Previous studies have shown flagellar involvement in the phage infection process. Phages that use the flagella as part of their infective process are referred to as flagellotropic and have been recently reviewed [29,30]. For flagellotropic phages, the flagella provide the initial binding followed by binding to a secondary surface receptor with resulting infection. In the case of bacteriophage 7-7-1, which infects *Agrobacterium* sp. H13-3, primary attachment occurs to the flagellar filament followed by delivery to the cell surface and interaction with a second receptor; in this case, LPS [31]. In contrast, ΦV10 as shown in this study, appears to initially bind to the O157 antigen (LPS) as the primary receptor. However successful infection after initial attachment as determined by both plaque and lysogen assays only occurs in the presence of flagella expressing the H7 antigen. Although the role of the H7 expressing flagella for infection was not elucidated in this study, its requirement for infection was demonstrated. This study was limited and based on phenotypic outcomes as described above; additional studies will be necessary to fully establish the mechanisms by which both the O157 and H7 antigens function to achieve bacteriophage binding and DNA uptake for successful infection by ΦV10.

## Figures and Tables

**Table 1:** Examples of various detection methods for *E. coli* O157:H7.

Method	Technique	Detection Target	Source
Chemiluminescence	Chemiluminescence	*rfbE* and *fliC*	[7]
Colorimetric	Nanozyme—mannose-modified Prussian blue nanoparticle recognition agent	*E. coli* O157:H7 flagella	[8]
Colorimetric	Wax-printed paper-based enzyme-linked immunosorbent assay	*E. coli* O157:H7 cells	[9]
Fluorescence labeling	Carbon quantum dot-magnetic nanoparticle probes	*E. coli* O157:H7 cells	[10]
Label-dependent electrochemical detection	Linear sweep voltammetry	Gold-binding peptides and alkaline phosphatase release from cells infected with an engineered T7 phage	[11]
Label-free electrochemical detection	Cyclic voltammetry	*E. coli* O157:H7 cell capture	[12]
Nanomaterial photothermal effect	Photothermal	*E. coli* O157:H7 cells	[13]
Nucleic acid	Quantitative polymerase chain reaction	*XanQ*	[14]
Nucleic acid	Recombinase-aided amplification	*fliC*	[15]
Optical scattering	Bacterial rapid detection using optical scattering technology	Colony morphology	[16]
Optical scattering	Gold nanoflowers used in dynamic light scattering	*E. coli* O157:H7 cells	[17]
Optical scattering	Gold nanobones used in surface-enhanced Raman scattering	*E. coli* O157:H7 cells	[18]

**Table 3:** ΦV10 plaque formation with *E. coli* O157:H7 strain C7927 and *E. coli* O157:H^−^ strain 493-89.

*E. coli* Strain	Number of Plaques at Different Phage Dilutions (PFU)							
	10^−2^	10^−3^	10^−4^	10^−5^	10^−6^	10^−7^	10^−8^	10^−9^
C7927	TMC *	TMC	TMC	TMC	410	48	2	0
493-89	0	0	0	0	0	0	0	0

* TMC—Too Many to Count.

**Table 4:** Lysogen and plaque formation by *E. coli* O157:H7 C7927, *E. coli* O157:H^−^ 493-89, *E. coli* O157:H16 P11, and *E. coli* O55:H7 1880. ΦV10*nluc* was used in lysogen assays, while wild-type ΦV10 was used in plaque assays.

*E. coli* Strain/Serotype	Phage Binding	Lysogen Formation	Plaque Formation
C7927/O157:H7	+	2.4 × 10^5^	+
1180/O55:H7	−	0	−
P11/O157:H16	+	0	−
493-89/O157:H^−^	+	2.4 × 10^3^	−
493-89 pFSP182/O157:H7	+	ND *	+

* ND—Not Determined.

**Table 5:** Plaque assay results of *E. coli* O157:H7 C7927, *E. coli* O157:H^−^ 493-89, *E. coli* O157:H^−^ 493-89 flhDC5-3, and *E. coli* O157:H^−^ 493-89 pFSP182 exposure to ΦV10.

*E. coli* Strain/Serotype	Motility	Plaque Formation
C7927/O157:H7	+	+
493-89/O157:H^−^	−	−
493-89 *flhDC*/O157:H7	+	−
493-89 pFSP182/O157:H7	+	+

## Data Availability

The original contributions presented in this study are included in the article. Further inquiries can be directed to the corresponding author.
